# Beyond first sip: combination of intermittent cow’s milk exposure and atopic dermatitis associated with severe IgE mediated cow’s milk reactions—a retrospective study

**DOI:** 10.1186/s13223-026-01035-z

**Published:** 2026-04-17

**Authors:** Amy Plessis, Victoria E. Cook, Stephanie C. Erdle, Edmond S. Chan

**Affiliations:** 1 Department of Pediatrics, University of British Columbia, Vancouver, BC Canada; 2 Division of Allergy, Department of Pediatrics, University of British Columbia, Vancouver, BC Canada; 3 BC Children’s Hospital Research Institute, Vancouver, BC Canada; 4 Community Allergy Clinic, Victoria, BC Canada

**Keywords:** IgE-mediated cow’s milk allergy, Intermittent cow’s milk formula, Atopy, Anaphylaxis, Prevention

## Abstract

**Background:**

The prevalence of IgE-mediated cow’s milk allergy in Canada has been increasing and is recognized as the leading cause of fatal anaphylaxis in school aged children. Experts are unable to predict which individuals will have spontaneous resolution of cow’s milk allergy and who will have a persistent phenotype, therefore primary prevention is key.

**Objective:**

The aim of this study was to identify risk factors leading to the development of an IgE mediated cow’s milk allergy.

**Methods:**

A 54-item survey was provided to parents of children aged 0–4 years with an allergist diagnosed IgE mediated cow’s milk allergy.

**Results:**

Out of the 24 participants who completed the survey, 21 were used in the analysis. All infants were term and 57% of participants reported intermittent exposure to cow’s milk formula. Of the infants with intermittent cow’s milk exposure, majority (75%) experienced the exposure prior to hospital discharge. Infants with both atopic dermatitis and intermittent exposure to cow’s milk formula had more severe reactions.

**Conclusion:**

This is the first study to our knowledge that found an association between reaction severity and having multiple risk factors- atopic dermatitis and intermittent exposure to cow’s milk formula. Majority of the intermittent cow’s milk exposure occurs prior to discharge from hospital. Further research in the area is required, however policy changes to mitigate associated risk could include use of donor breast milk or extensively hydrolyzed formula for supplementation, or recommendations to continue regular cow’s milk formula following introduction.

**Supplementary Information:**

The online version contains supplementary material available at 10.1186/s13223-026-01035-z.

## Clinical implications

IgE mediated cow’s milk allergy is the leading cause of fatal food related anaphylaxis in children. This article highlights potential key risk factors in development of an IgE mediated cow’s milk allergy and policy changes for prevention.

The prevalence of IgE-mediated allergy to cow’s milk (IgE-CMA) in Canada has been increasing, with a reported prevalence of around 1.2% [1]. In the United Kingdom, IgE-CMA is the leading cause of fatal anaphylaxis in schoolchildren [2]. While there is a high rate of spontaneous resolution of IgE-CMA with 53–57% of children outgrowing IgE-CMA by age 5 and 72% by age 17, the cases that persist cannot be reliably predicted [3]. Allergy prevention is therefore critical. While sound randomized control trials on risk factors leading to the development of IgE-CMA are lacking, there is evidence that intermittent exposure to cow’s milk formula (CMF) during infancy is a risk factor for IgE-CMA and severity of reaction has been associated with a more persistent phenotype [4]. Existing studies, however, often exclude preterm infants and data on the risk factors for IgE-CMA remains limited.

We provided a 54-item survey to parents of children 0–4 years of age with allergist-diagnosed IgE-CMA with both a history of IgE-mediated symptoms following cow’s milk (CM) ingestion and positive skin prick testing (SPT) or serum specific-IgE to CM (ssIgE-CM). Reactions were considered severe if they were classified as grade 2 or higher. This study did not warrant formal ethics review, as the University of British Columbia categorized it as quality improvement.

Between July 2023–May 2025, 24 participants completed the survey. Three patients were excluded; one patient did not meet the age cutoff, and two reported reactions not consistent with IgE-CMA.

All participants were term infants, and none spent time in the neonatal intensive care unit (NICU). The median SPT from 18 patients was 9.25 mm (IQR = 4), 4 patients had ssIgE-CM (13, 2.48, 88.3, 20 (KU/L)). Twelve (57%) participants reported intermittent exposure to CMF. Nine (43%) were exposed to CMF prior to leaving the hospital, and 3 (14%) received CMF after hospital discharge. The most common reasons for hospital supplementation were insufficient breast milk supply (4/9), jaundice (3/9) and hypoglycemia (2/9). None of the infants continued CMF beyond 3 months of age. One infant transitioned to extensively hydrolyzed formula within 1 month of age (1/10).

**Fig. 1:**
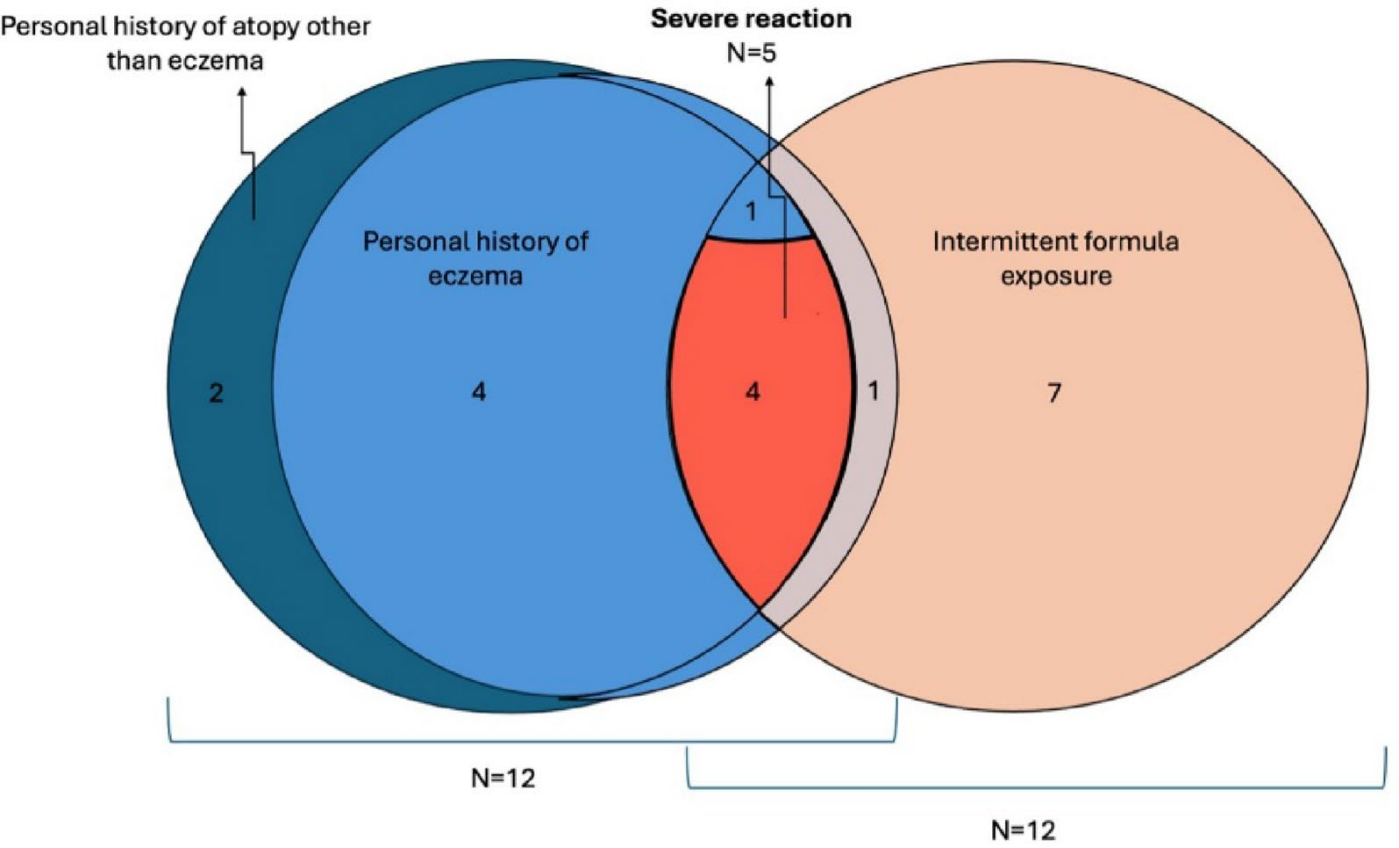
Venn diagram illustrating the distribution of infants (*N* = 21) based on intermittent CMF exposure, personal history of atopy and severe reaction. Among the five infants who had both a personal history of atopy and intermittent CMF exposure, four had a severe reaction

At the time of survey completion, a history of atopy was reported in over half of the infants, 12/21 (57%) and 11/21 (52%) had a first degree relative with a history of atopy. The most common reported atopic condition in the infants was atopic dermatitis (AD) (10/21, 48%) (Fig. 1). Out of the 9 children without atopic disease, 7 had CMF exposure. There were only two children without a personal history of atopy or CMF exposure.

When looking at reaction severity there were only 5 infants who met criteria for anaphylaxis with two system involvement, and 3 of these infants received epinephrine, with 2/3 requiring multiple doses. The remainder of the infants had isolated cutaneous symptoms. There were 5/21 (24%) infants who had both CMF exposure in the first 2 weeks of life and AD. All but one of these infants stopped formula by 2 weeks of life; the one that continued with formula was switched to hydrolyzed formula. Of the infants with anaphylaxis, 4/5 (80%) of them fell into the category of having both a history of AD and CMF exposure in the first 2 weeks of life with SPT ≥ 6 mm (Fig. 1). The 1 other infant with anaphylaxis had AD but no history of CMF exposure and SPT 3.4 mm (Fig. 1).

This is the first study to our knowledge that allowed for the inclusion of preterm infants and infants in the NICU. Despite this, none of the participants fell into these categories. The rate of preterm births in Canada is 8%, therefore we would have expected to capture 2 infants at minimum in our sample [5]. NICU admission is associated with a higher rate of exposure CM products, with 71% of infants exposed to human milk fortifiers, primarily Bovine-derived, and 48% exposed to CMF [6]. These rates are higher than those seen in healthy term infants, where 19% require formula supplementation in the first 2 days of life [7]. Supplementation with CMF is a risk factor for development of IgE-CMA for term infants; however, preterm infants would have much higher rates of exposure to CMF shortly after birth, yet do not seem to have higher rates of IgE-CMA [4, 8]. It is most likely that preterm infants continue to maintain regular ingestion of CMF, which is protective against development of IgE-CMA, as only 33% of infants born at 32–36 + 6 gestation are discharged on exclusive breastmilk [9]. Alternative explanations include differences in the developing immune system that do not result in the same IgE sensitization to CM as the term infant [10]. Further research into risk factors for the development of IgE- CMA in the preterm population is needed.

Our results are consistent with other studies that show that intermittent CMF exposure is a risk factor for development of IgE-CMA, with 57% of infants in our sample having intermittent CMF exposure and 75% of the CMF exposed group being prior to hospital discharge [4, 8]. Consistent with the literature, other risk factors for development of IgE-CMA noted in our cohort was AD, which was present in 48% of the infants. Individuals with both risk factors (AD and intermittent CMF exposure) seemed to have more severe reactions compared to individuals with just one risk factor. The majority (4/5, 80%) of the children with a severe reaction had both risk factors; only one child with both risk factors had a mild reaction. The 4 infants with both risk factors also had a SPT ≥ 6 mm, which could indicate a more persistent form of IgE-CMA [3]. This is the first description of individuals with both these risk factors and severity of reaction.

Limitations of this study include the retrospective design which could lead to recall bias, the small sample size, lack of diverse risk factors, and lack of a control group.

Both AD and intermittent CMF exposure are independent risk factors for development of IgE-CMA according to prior studies [3, 4]. Our data suggests individuals with both risk factors may have more severe reactions, although more research is required. The majority of CMF exposure occurs prior to hospital discharge for healthy term neonates. More randomized controlled trials looking at early and intermittent CMF supplementation are needed, however if shown to be a consistent risk factor then policy changes to mitigate associated risk could include use of donor breast milk or extensively hydrolyzed formula for supplementation, or recommendations to continue regular CM following introduction.

## Supplementary Information

Below is the link to the electronic supplementary material.

Supplementary Material 1

## Data Availability

All data generated or analyzed during this study are included in this published article. The original dataset is available through the corresponding author on reasonable request.

## Abbreviations

IgE-CMA: IgE-mediated allergy to cow’s milk
CMF: Cow’s milk formula
CM: Cow’s milk
SPT: Skin prick testing
ssIgE-CM: Serum specific-IgE to cow’s milk
NICU: Neonatal intensive care unit
AD: Atopic dermatitis
