# Supplementary figures and images for: Beyond first sip: combination of intermittent cow’s milk exposure and atopic dermatitis associated with severe IgE mediated cow’s milk reactions—a retrospective study
Source: Allergy Asthma Clin Immunol. 2026 Apr 17;22:20. doi: 10.1186/s13223-026-01035-z (PMC13088481; doi:10.1186/s13223-026-01035-z)

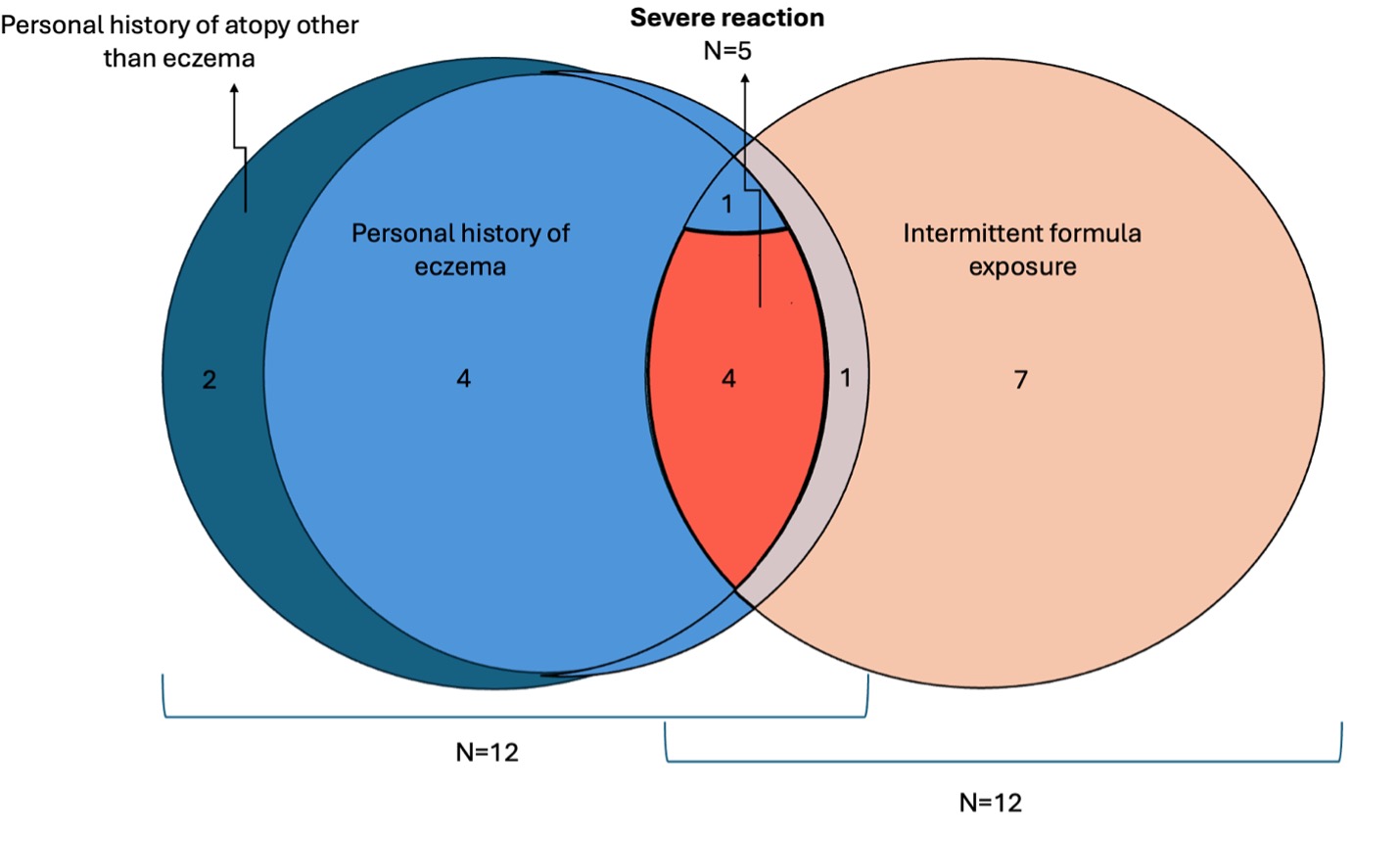

Supplement: Supplementary file 1 — Supplementary Material 1 [file 13223_2026_1035_MOESM1_ESM.jpg]
